# A Novel Use of Next-Generation Closed Incision Negative Pressure Wound Therapy After Major Limb Amputation and Amputation Revision

**DOI:** 10.7759/cureus.10393

**Published:** 2020-09-11

**Authors:** Julie West, Julia Wetherhold, Steven Schulz, Ian Valerio

**Affiliations:** 1 Plastic and Reconstructive Surgery, The Ohio State University Wexner Medical Center, Columbus, USA; 2 Plastic and Reconstructive Surgery, Massachusetts General Hospital, Boston, USA

**Keywords:** major limb amputation, incisional negative pressure wound therapy, closed incisional negative pressure wound therapy, surgical site infection, wound healing, post-operative management, amputation revision, residual limb pain, targeted muscle reinnervation

## Abstract

We report our experience with next-generation incisional negative pressure wound therapy (iNPWT) applied after major limb amputation or amputation revision. In this high-risk patient population, the need for reliable post-operative soft tissue management is imperative. In both cases reported, healing was uncomplicated. Using the next generation iNPWT in this unique way optimizes the post-operative residual limb by improved incision healing, residual limb edema reduction, and reduced risk of surgical site infection (SSI). This is the first case report of its kind reporting a novel use of next-generation iNPWT, and it demonstrates a need to examine this particular use further.

## Introduction

Major extremity amputation is a life-altering event. In the United States, there are an estimated 2 million Americans living with limb loss, and this figure is projected to double by the year 2050 [1]. The impact of a major limb amputation is significant in both the acute and long-term settings, and effective post-operative incision management is a critical first step. In the acute post-operative period, amputation site healing can be complicated by surgical site infections (SSI) and wound dehiscence, each posing significant risks to patients and the healthcare system [2]. These complications may lead to prolonged hospitalization and/or readmission, which in turn may increase healthcare costs and apply stressors to patients and their support systems [3]. Furthermore, wound healing complications in the early post-operative period for an amputee often lead to increased pain and opioid use that may predispose patients to chronic dependence [4]. In fact, as many as 95% of patients experience one or more types of amputation-related pain long term. Additionally, delays in early rehabilitation and prosthetic fitting caused by incisional complications significantly influence the patient’s abilities to perform activities of daily living (ADLs) and return to work [5]. Thus, reducing the risk of post-operative complications is of utmost importance in this patient population [6,7].

Incisional negative pressure wound therapy (iNPWT) has been well established in the literature as an effective adjunct for postoperative closed incision management and reduced SSI rates in patients at high risk for surgical site complications [2]. Patient comorbidities (e.g. obesity, diabetes) also adversely affect incisional healing [8]. In a 25 patient retrospective review, the authors reported successful institutional experience with iNPWT placed at the time of major limb amputation or amputation revision. No patients developed dehiscence, seroma, or hematoma and one patient developed an SSI that was treated outpatient with oral antibiotics [9]. Recently, second-generation iNPWT has been introduced to help further protect the incision by optimizing the management of the larger soft tissue envelope. Here, we report the novel application of the PREVENA RESTOR BELLA•FORM™ System (KCI, 3M company, San Antonio, TX) at the time of major limb amputation and amputation revision.

## Case presentation

Case 1

**Figure 1:**
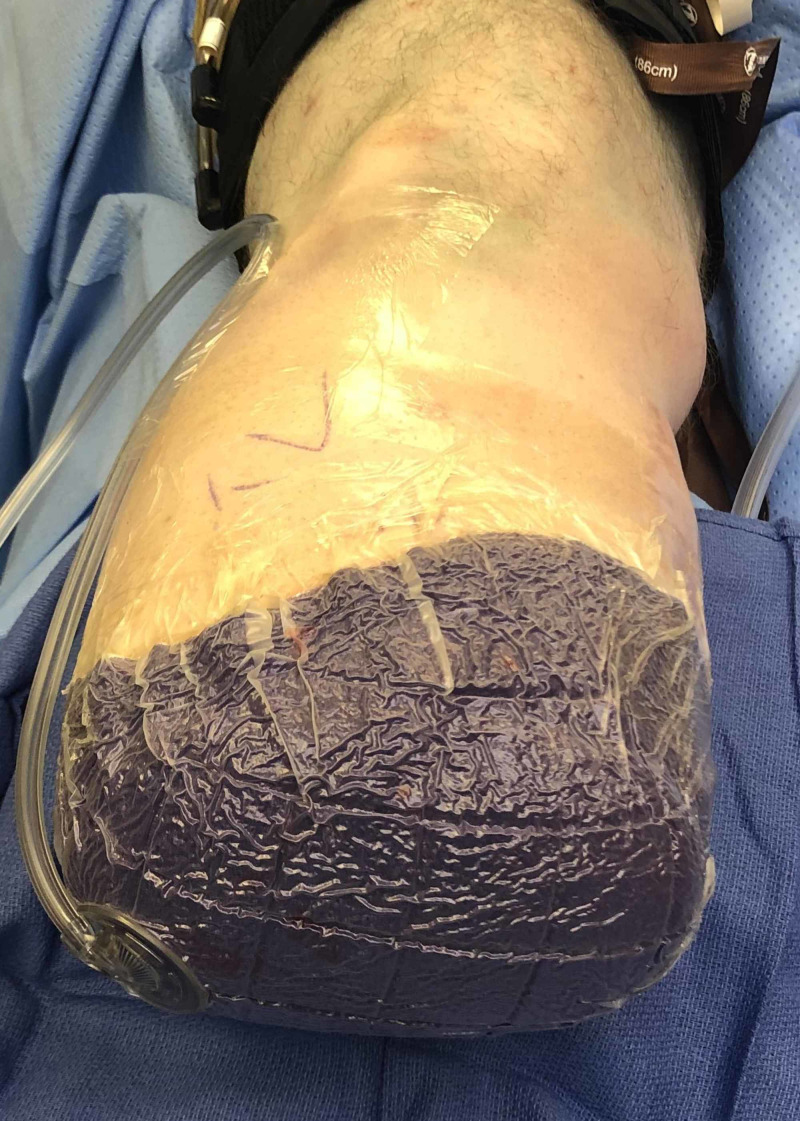
Intra-Operative Application of iNPWT after a Below Knee Amputation iNPWT = Incisional Negative Pressure Wound Therapy

**Figure 2:**
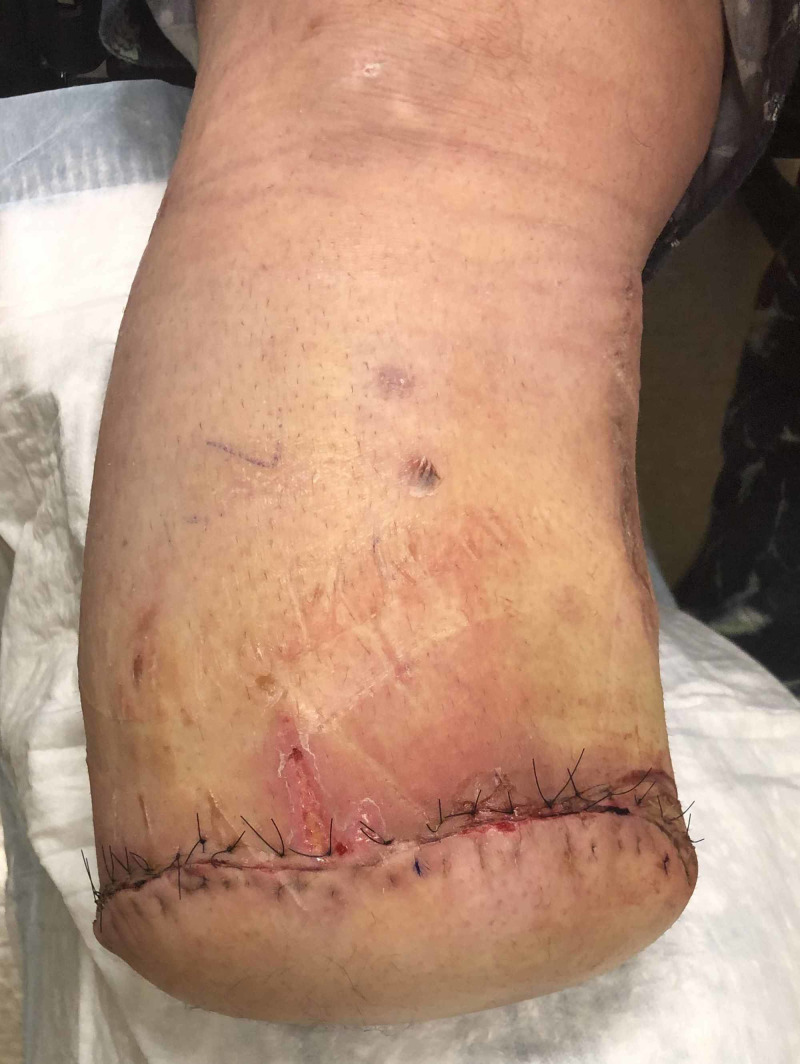
Intact Incision after iNPWT Removal on Post-Operative Day 9 iNPWT = Incisional Negative Pressure Wound Therapy

A 67-year-old male with multiple medical comorbidities presented with an infected open wound with acute osteomyelitis of his right partial foot. The patient underwent an aortic valve replacement complicated by aortic rupture that ultimately was successfully repaired. During his post-operative recovery, he developed heparin-induced thrombocytopenia (HIT); he was critically ill and subsequently developed vasopressor-induced necrosis of his hands and feet. Over time, his hand necrosis resolved, but he went on to develop dry gangrene of both feet. His left foot became infected and he underwent a left below knee amputation (BKA) while his right foot was left to declare itself over time. He recovered uneventfully and began to ambulate in a prosthetic on the left side. Six months after continuing dry dressings and betadine paint to the dry gangrene on his right foot, he presented to our institution for evaluation for reconstructive options. He was hoping he had enough remaining foot to avoid another BKA. He was assessed and then underwent a right transmetatarsal amputation. The postoperative course was complicated by the development of a plantar foot wound near his surgical site. The patient went on to develop acute osteomyelitis throughout his residual foot. As a result, the patient underwent a right BKA with targeted muscle reinnervation (TMR) with application of a PREVENA RESTOR BELLA•FORM™ System set to 125 mmHg continuous suction. The right BKA was performed by orthopedic surgery with careful attention to ensure that the tibial length closely mirrored that of his left BKA and a beveled cut was made 15 cm from the tibial tubercle. TMR was performed at the time of amputation to decrease the risk of neuroma formation and phantom limb pain. The patient was placed in a knee immobilizer and was evaluated post-operatively by physical therapy. He returned home and the iNPWT was removed after nine days when he returned to the clinic. His soft tissue edema was well controlled and the incision healed without complication (Figures 1-2)

Given his comorbidities and prior wound healing difficulties, the patient progressed through the post-operative period without complication. He transitioned into a residual limb compression sleeve as directed by his prosthetist and eight weeks after amputation, he began wearing his prosthetic. At his six-month follow-up, the patient remains healed, with no residual limb or phantom limb pain, and ambulates daily with a cane in his bilateral below-knee prosthetics.

Case 2

**Figure 3:**
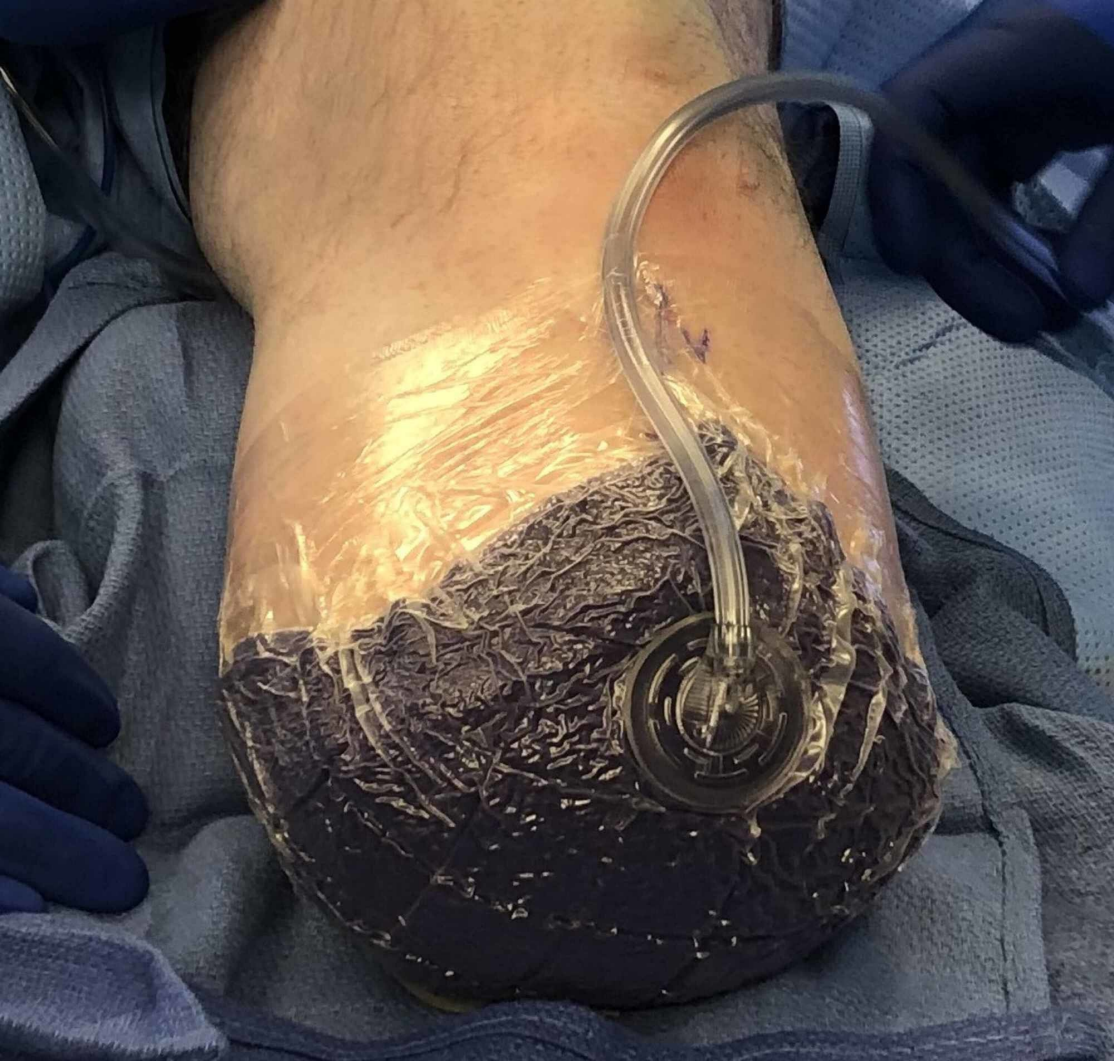
Intra-Operative Application of cINPT after a Below Knee Amputation Revision cINPT = Closed Incisional Negative Pressure Wound Therapy

**Figure 4:**
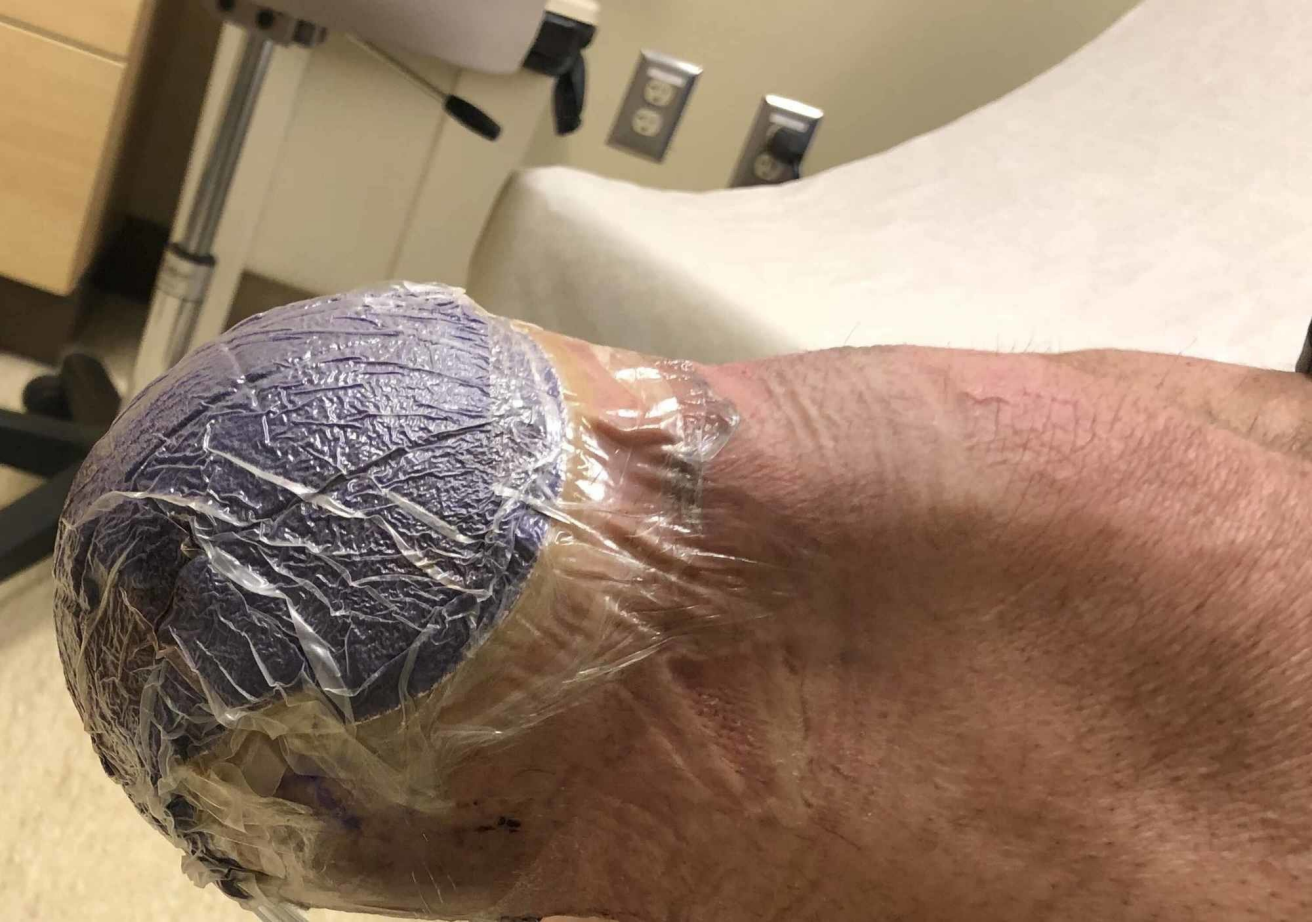
Intact cINPT after Below Knee Amputation Revision Prior to Removal cINPT = Closed Incisional Negative Pressure Wound Therapy

A 40-year-old male with a history of type 1 neurofibromatosis who had undergone a left BKA with TMR and iNPWT placement one year prior, presented with significant soft tissue changes in his residual limb rendering him unable to wear his prosthetic. His prosthetist had performed socket modifications and he was using multiple layers of socks to keep on the prosthesis. He underwent a left BKA stump soft tissue revision with application of a PREVENA RESTOR BELLA•FORM™ System. The closed incisional negative pressure wound therapy (cINPT) was removed on post-operative day 14 and the patient noted a pain-free removal. The incision was well-healed with minimal soft tissue edema (Figures 3-4).

His soft tissue remained stable and he transitioned into a residual limb compression sleeve. His prosthetist was able to re-fit his socket and he began ambulating at the 6-week mark.

## Discussion

When facing a major extremity amputation, the post-operative care is often difficult and complex for patients to navigate. The majority of patients undergoing a major extremity amputation or amputation revision fall into the high-risk category [8]. High-risk classification is associated with many factors including procedure performed and patient comorbidities. Factors that commonly contribute to an increased mortality for this specific patient population include peripheral vascular disease, diabetes, infection, smoking history, and re-operation and/or soft tissue injuries [10]. Early post-operative wound complication rates vary from 12% to 34% in patients undergoing a BKA and the incidence of wound complications increases the length of post-operative hospitalization [10,11]. Our previously published retrospective cohort study involving iNPWT and amputees demonstrated that use in the immediate postoperative period aids in the management of these challenging incisions with only a 4% rate of superficial wound complications, all managed in the outpatient setting [9]. Effective initial wound management in this patient population helps reduce hospital length of stay and expedites time to rehabilitation and prosthetic fitting which may ultimately contribute to an improved quality of life.

iNPWT was designed to manage post-operative incisions. Over time, it has proven to be an effective therapy for a number of reasons. It acts as a soft tissue splint to reduce tension on the incision line, which allows for improved healing and decreased dehiscence rates [12,13]. The continuous nature of iNPWT has been shown to decrease post-operative tissue edema and ultimately seroma and hematoma formation [9,14]. Recently, the Food and Drug Administration (FDA) published de novo guidelines stating that the use of iNPWT reduces the incidence of seroma formation and in high-risk patients, it can aid in the reduction of SSI formation.

Next-generation iNPWT has been developed to not only manage post-operative incisions, but to impact the soft tissue envelope around the surgical site. The larger surface area of these dressings helps not only promote incision healing by reducing incisional tension, but also to reduce edema to the surrounding soft tissue that occurs following surgery. The early effectiveness of this device has been primarily demonstrated in breast surgery patients; however, this may have an important role in the post-operative amputee population. Our successful early experience with PREVENA RESTOR BELLA•FORM™ System from a provider standpoint included ease of application and the ability to conform to various soft tissue landscapes, especially following extremity amputation. For the patients, ease of removal, edema reduction in their soft tissue envelope, and improved incision healing are important factors in the decision to use this. The larger dressing size allows it to conform to the end of the residual limb, not just the incision line, and iNPWT over this larger surface area improves lymphatic drainage thereby reducing soft tissue edema. Additionally, this reduced the need for additional immediate post-operative compression as more surface area on the residual limb was covered. 

This case report presents two patients and their outcomes using next-generation iNPWT. We believe that there may be added benefits as compared to traditional iNPWT in both comfort during use and removal and edema control of the residual soft tissues. Future studies comparing traditional iNPWT, next-generation iNPWT, and no iNPWT are warranted to further improve our understanding as well as specific indications for when to use iNPWT in the amputee patient population.

## Conclusions

Post-operative iNPWT is an effective way to minimize wound complications after major extremity amputation or amputation revision. This is the first case report of its kind reporting a novel use of next-generation iNPWT to optimize the post-operative residual limb. Next-generation iNPWT was safely utilized in these two cases and shows promise for this at-risk population. Further prospective studies are needed.
